# Delayed acquisition of *Plasmodium falciparum* antigen-specific CD4^+^ T cell responses in HIV-exposed uninfected Malawian children receiving daily cotrimoxazole prophylaxis

**DOI:** 10.1186/s12936-016-1318-2

**Published:** 2016-05-10

**Authors:** Herbert Longwe, Kamija S. Phiri, Nyanyiwe M. Mbeye, Thandile Gondwe, Wilson L. Mandala, Kondwani C. Jambo

**Affiliations:** Department of Basic Medical Sciences, College of Medicine, University of Malawi, Blantyre, Malawi; Tropical Haematology Research Unit, College of Medicine, University of Malawi, Blantyre, Malawi; Department of Public Health, College of Medicine, University of Malawi, Blantyre, Malawi; Malawi-Liverpool-Wellcome Trust Clinical Research Programme, Blantyre, Malawi; Liverpool School of Tropical Medicine, Liverpool, UK

**Keywords:** *Plasmodium falciparum*, Cotrimoxazole, HIV-exposed children, CD4^+^ T cells

## Abstract

**Background:**

Cotrimoxazole (CTX) prophylaxis, recommended in HIV-exposed uninfected (HEU) children primarily against HIV-related opportunistic infections, has been shown to have some efficacy against *Plasmodium falciparum* malaria. The effects of CTX prophylaxis on the acquisition of *P. falciparum* antigen specific CD4^+^ T cells-mediated immunity in HEU children is still not fully understood.

**Methods:**

Peripheral blood was collected from HEU and HIV-unexposed uninfected (HUU) children at 6, 12 and 18 months of age. Proportion of CD4^+^ T cells subsets were determined by immunophenotyping. *P. falciparum* antigen-specific CD4^+^ T cells responses were measured by intracellular cytokine staining assay.

**Results:**

There were no differences in the proportions of naïve, effector and memory CD4^+^ T cell subsets between HEU and HUU children at all ages. There was a trend showing acquisition of *P. falciparum*-specific IFN-γ and TNF-producing CD4^+^ T cells with age in both HUU and HEU children. There was, however, lower frequency of *P. falciparum*-specific IFN-γ-producing CD4^+^ T cells in HEU compared to HUU at 6 and 12 months, which normalized 6 months after stopping CTX prophylaxis.

**Conclusion:**

The results demonstrate that there is delayed acquisition of *P. falciparum*-specific IFN-γ-producing CD4^+^ T cells in HEU children on daily cotrimoxazole prophylaxis, which is evident at 6 and 12 months of age in comparison to HUU age-matched controls. However, whether this delayed acquisition of *P. falciparum*-specific IFN-γ-producing CD4^+^ T cells leads to higher risk to malaria disease remains unknown and warrants further investigation.

## Background

In children, repeated exposure to *Plasmodium falciparum* antigens contributes towards the development of effective immunity that controls parasitaemia and prevents severe and life threatening illness [1, 2]. Evidence from mouse models and experimental human infections suggests that both CD4^+^ and CD8^+^ T cells play an important role in protective immunity to *P. falciparum* malaria [3–6]. T cells directly control the development of pre-erythrocytic *Plasmodium* stages and also direct the immune responses induced by erythrocytic stages of malaria [7]. During blood stage infection, acute pro-inflammatory cytokine-mediated effector responses from CD4^+^ T cells can restrict growth of the malaria parasites and reduce risk of clinical disease [7]. Of note, production of IFN-γ, TNF, IL-2, IL-6 and IL-4 has been shown in various ways to play a role in anti-malarial immunity [6, 8–13]. IFN-γ has been proposed as the major mediator of parasite clearance during blood stage infection [14].

Continuous malaria chemoprophylaxis has been shown to slow down the development of clinical immunity in children leading to an increased risk of disease following disruption or cessation of treatment [15, 16]. Cotrimoxazole (CTX), a potent antibiotic and with strong anti-malarial properties [17], is recommended for prophylaxis in HIV exposed uninfected (HEU) children in the first 12 months of age, primarily to prevent HIV-related opportunistic infections. Due to its anti-malarial properties, CTX may potentially confer malaria chemoprophylaxis, preventing the natural exposure to *P. falciparum* and subsequent acquisition of malaria specific immunity.

Although there is now good evidence to show that CTX prevents clinical malaria in children taking it [17, 18], limited data exist on the impact of CTX exposure on development of clinical immunity to malaria following cessation of prophylaxis. Immunological measurements against malaria antigens may provide a useful approach to monitor development of natural immunity to malaria during and following termination of CTX prophylaxis. Of the few studies that have looked at the effect of malaria chemoprophylaxis on both humoral and cellular immune responses during and post prophylaxis, the results have been somehow contradictory with children who had received chemoprophylaxis showing reduced levels of malaria specific antibodies but increased cell mediated immunity [19, 20]. Contrary to studies by Aponte et al. [15] and Menendez et al. [16], these earlier studies [19, 20] showed no lasting impairment of the protective immunity as measured by incidence of clinical disease in the year following cessation of chemoprophylaxis indicating that perhaps cell mediated immunity is protective despite lower antibody response.

Since CTX is given to these HEU children primarily as an antibiotic rather malaria chemoprophylaxis, its impact on development of natural immunity to malaria in HEU children needs careful assessment. HEU Malawian children on CTX prophylaxis have reduced magnitude and breadth of IgG antibody responses to distinct *P. falciparum* merozoite antigens [21] indicating a lack of exposure to malaria antigens. Given the importance of T cell mediated immunity to malaria and that T cell response to malaria antigens correlates poorly with humoral immune responses [19, 22], this study investigated the effects of CTX prophylaxis on the magnitude of *P. falciparum* antigen specific CD4^+^ T cells in HEU children during and after prophylaxis, and compared with aged-matched HIV-unexposed uninfected (HUU) children.

## Methods

### Study population

Characteristics of the study area, design and participants have already been described in detail before [21]. Briefly, the study was conducted in Zomba district, Malawi where *P. falciparum* malaria transmission is stable throughout the year, with *P. falciparum* prevalence rate of around 27 % [23]. HEU children were recruited from the ART clinic at the Zomba Central Hospital and aged matched HUU children as controls from the communities where HEU children were based. Children who were classified as healthy at the time of recruitment and were not on any medication and were breastfeeding were included in the study. As a national policy for Malawi, daily CTX prophylaxis is given to all HEU children from 6 weeks of age, and all pregnant mothers are put on antiretroviral therapy (ART). Individual written informed consent was obtained from the parents or guardians of all children. The College of Medicine Research Ethics Committee (COMREC) approved this protocol.

### Collection and processing of samples

Children were seen every 6 months and at each visit, a total of 3 ml of venous blood was collected. 2 ml in sodium heparin tube for intracellular cytokine assay and 1 ml of in EDTA tubes for immunophenotyping. All immunological assays were conducted blinded to the children’s study group.

### *Plasmodium falciparum* antigens

Cryopreserved laboratory adopted strain of *P. falciparum* (3D7) was kindly provided by ICMER laboratory, Blantyre, Malawi. For recovery, parasite aliquots were thawed using two sodium chloride thawing solutions: (200 µl of 12 % NaCl and 10 ml of 1.6 % NaCl) and washed twice with 25 ml of protein free medium (RPMI-1640 supplemented with 25 mmol/l HEPES and 40 mg/ml gentamycin), as described elsewhere [24]. Parasite culture to late trophozoite stage was done using standard protocols as previously described [25].

Level of parasitaemia was estimated based on microscopy of thin smears and parasitaemia level of approximately 5 % was used. Pooled cultures were washed and 1.5 ml of warm protein free medium was added to the red cell pellet followed by 2.5 ml warm gelofosine (Bbraun, Melsungen, Germany). The mixture was then allowed to stand for 20 min at 37 **°**C and supernatant carefully removed and transferred to a second tube. The mixture was washed and approximately 1 μl of the pellet was collected and parasitaemia determined as previously described [25]. The infected erythrocytes were lysed using saponin lysis procedure as previously described [26]. Protein content was determined by NanoDrop as per manufacturer’s instructions and the lysate stored at −80 **°**C.

### Immunophenotyping

Whole blood (20 μl) was stained with surface markers (CD3 PE, CD4 PerCP and CD8 APC-H7 (all from BD Biosciences, San Jose, CA, USA), CD45RA FITC (BD Pharmingen, San Jose, CA, USA) and CCR7 APC (eBiosciences, San Diego, California) and lysed with 2 ml of 1× FACS lysing solution. After wash, the cells were then acquired on a Cyan ADP flow cytometer (Beckman Coulter) and minimum of 10,000 lymphocytes events was collected per tube during acquisition.

### Intracellular cytokine staining

Heparinized whole blood (100 μl) was cultured in 96 U bottom culture plate and incubated with PMA/Ionomycin (10 ng/ml and 100 µg/ml, Sigma-Aldrich, UK), *P. falciparum* 3D7 schizonts lysate (5 µg/ml) and mycobacterium purified protein derivative (10 µg/ml, Statens Serum Institut, Denmark) in parallel with a non-stimulated background control for 24 h. Harvested cells were stained with surface markersCD3-APC-H7, CD4-PerCP (all from BD Biosciences, San Jose, CA, USA), and CD8-Pacific Orange (Invitrogen™ Life technologies, Carlsbad, CA, USA) and lysed as above. The cells were the permeabilized with 500 μl of 1× BD Cytofix/Cytoperm (BD Cytofix/Cytoperm™, San Diego, California) and then stained with intracellular markers IFN-γ-FITC (BD FastImmune™, San Jose, CA, USA) and TNF-PE (BD Biosciences, San Jose, California) for 30 min. The cells were then acquired as above and minimum of 10,000 CD4^+^ T cell events were collected per sample. Single stained BD comp beads were processed and acquired in parallel to samples each day and used for compensation. Analysis was conducted using FlowJo software version 9.7.5 (Tree Star Inc., San Carlos, CA, USA).

### Statistical analysis

Statistical analysis and graphical presentation were done using GraphPad Prism 6 (GraphPad, California, USA) and STATA (StataCorp, Texas, USA). A Shapiro–Wilk normality test revealed that all data were non-normally distributed. Mann–Whitney U test was used for comparison CD4^+^ T cell proportions and frequencies of cytokine producing cells between the study groups at each visit. Background responses generated in the negative control wells were subtracted from responses generated from antigen-stimulation to obtain antigen-specific T cell responses. Differences with *P* values less than 0.05 were considered significant.

## Results

### Characteristics of study participants

Overall, 33 HEU children and 31 age and location-matched HUU children were recruited in the study and followed through from 6 to 18 months of age (Table 1). Both the HEU and HUU groups were comparable for both baseline and follow up characteristics. All children were afebrile and asymptomatic at the time of venous blood draw at each visit. The median birth weight was similar between HEU and HUU children (3.2 vs. 3 Kg, *p* = 0.21). All mothers of the HEU children were on ART and were taking daily CTX prophylaxis during the entire follow up period.

**Table 1:** Demographic characteristics of the study participants

Description^a^	Age of study participants at each visit								
	6 months			12 months			18 months		
	HUU	HEU	p*	HUU	HEU	*p*	HUU	HEU	*p*
*Demographic data*									
Number of children per visit	31	33	–	29	31	–	28	30	–
Females (%)	65	46	–	62	42	–	64	40	–
*Laboratory parameters*									
WBC count, ×10^3^ cells/ml	10.2	9.45	*0.51*	11.5	12.0	*0.65*	9.5	8.4	*0.34*
Total CD4 T cells (%)	58.4	55.2	*0.042*	51.1	54.8	*0.62*	48.8	54.8	*0.052*
*Mothers health status*									
Mother on ART, %	–	97	–	–	100	–	–	97	–
Mother on CTX, %	–	100	–	–	100	–	–	100	–

*p* *p* value, *WBC* white blood cells, *ART* antiretroviral therapy, *CTX* cotrimoxazole

* p value obtained with Mann–Whitney U test, *p* value of <0.05 was considered significant

^***a***^Presented as medians

### Proportion of CD4^+^ T cell subsets in HEU and HUU children at 6 , 12  and 18 months

**Fig. 1:**
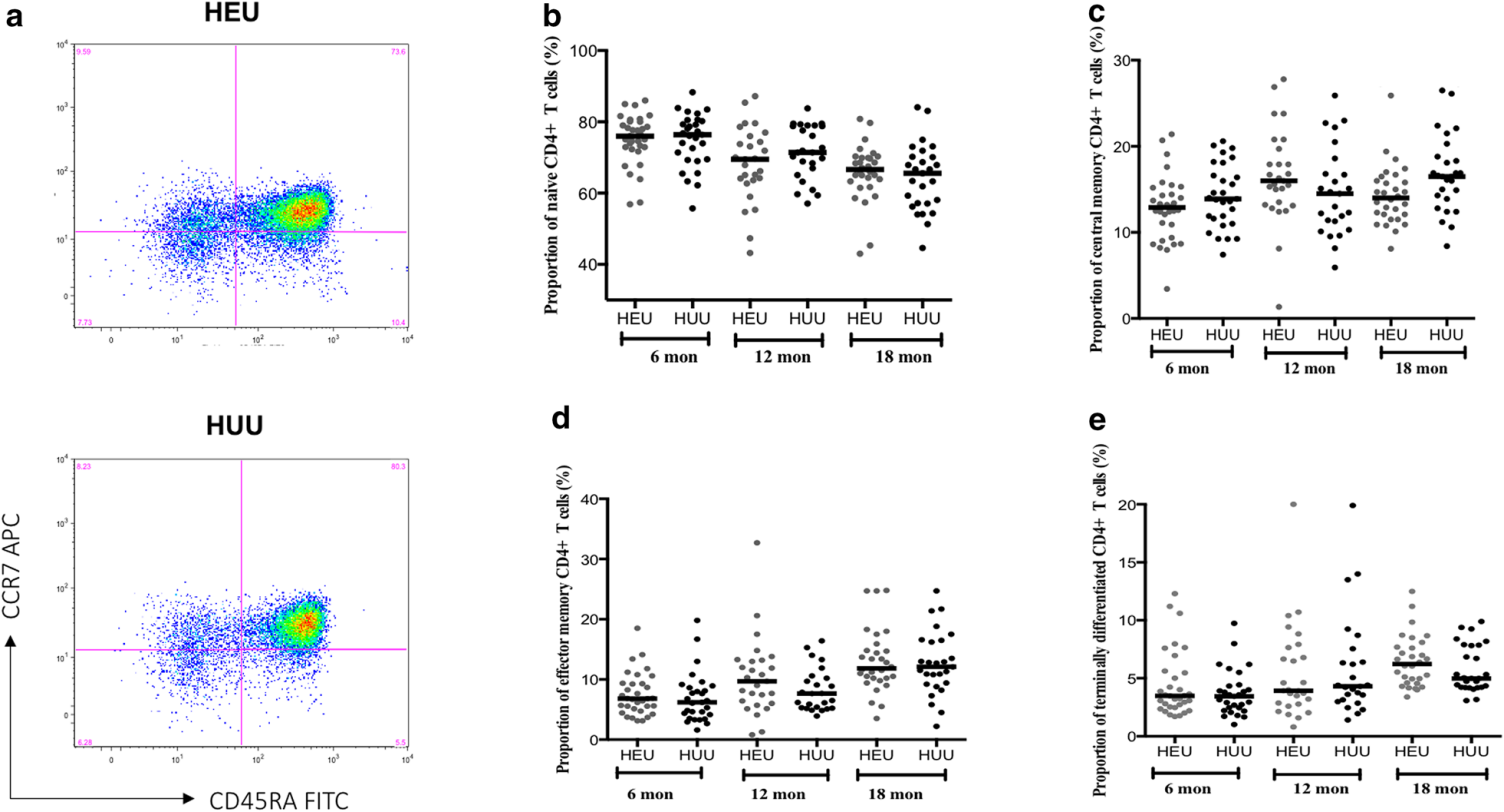
The proportions of naive and memory CD4^+^ T cell subsets in HEU and HUU children at different ages. Cells were stained anti CD3 PE, anti CD4 PerCP, anti CD8 APC-H7, anti CD45RA FITC and anti CCR7 APC monoclonal antibodies. **a** A gate was drawn on CD4^−^ CD8^−^(non T cells) and CD4^+^ CD8^−^ (CD4^+^ T cells) populations and plotted on CD45RA FITC against CCR7 APC. Using quadrant statistics, distinct populations of naïve (CD45RA^+^ CCR7^+^), central memory (CD45RA^−^CCR7^+^), effector memory (CD45RA^−^ CCR7^−^), and terminally differentiated (CD45RA^+^ CCR7^−^) CD4^+^ T cells were identified. Median proportions of Naïve CD4^+^ (**b**), central memory CD4^+^ (**c**), effector memory CD4^+^ (**d**), and terminally differentiated CD4^+^T lymphocytes (**e**) were determined in peripheral blood of HEU and HUU children at 6, 12 and 18 months. X-axis represents age of children in months. *Horizontal bars* represent medians

**Fig. 2:**
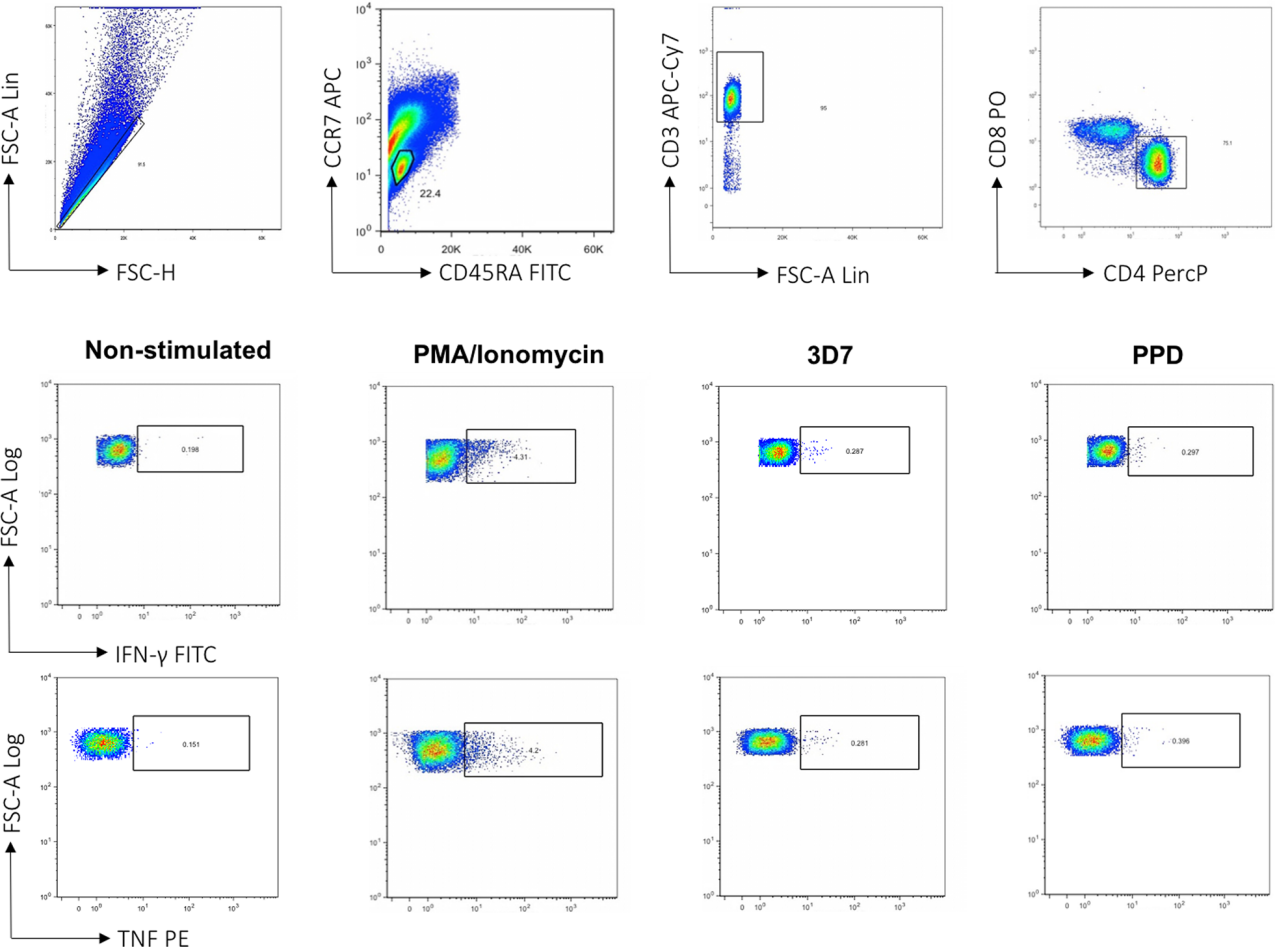
Representative FACS plots showing gating strategy for identifying antigen specific CD4^+^ T cell by intracellular cytokine staining assay. Heparinized whole blood collected at 6, 12 and 18 months was stimulated with PMA, *P. falciparum* 3D7 strain, purified protein derivative (PPD) and no antigen for 24 h. Lymphocytes were gated using FSC vs. SSC *plots*, FSC vs. CD3^+^ T cells and then CD4^+^T cells vs. CD8^+^T cells to isolate CD4^+^ T cells. Non-stimulated control samples were used to establish the threshold quadrants of background responses that were applied to quantify positive cytokine-producing cells in paired antigen-stimulated samples

Immune phenotypic defects have been previously reported in HEU infants, mostly in the pre-ART era [28], but it still remains unclear whether this is the case during the ART era. This study, therefore, phenotypically characterized CD4^+^ T cell subsets in HEU children and HUU aged-matched controls, to investigate whether HIV exposure and CTX prophylaxis alters the population of CD4 T cell subsets in children. The expression of CD45RA and CCR7 allowed for the characterization of naïve (CD45RA^+^CCR7^+^), central memory (T_CM_, CD45RA^−^CCR7^+^), effector memory (T_EM_, CD45RA^−^CCR7^−^), and terminally differentiated (CD45RA^+^CCR7^−^) T cells (Fig. 1a). There were no significant differences in the median proportion of naïve CD4^+^ T cells (Fig. 1b), T_CM_ CD4^+^T cells (Fig. 2c), T_EM_CD4^+^ T cells (Fig. 2d) and terminally differentiated CD4^+^ T cells (Fig. 2e) between HEU and HUU children at all ages (6, 12 and 18 months). These data suggest that HIV exposure or probably CTX prophylaxis did not lead to any apparent impairment in the populating of CD4^+^ T cell subsets beyond 6 months of age in HEU children.

### Frequencies of total cytokine-producing *Plasmodium falciparum* antigen specific CD4^+^ T cells in HEU and HUU children

**Fig. 3:**
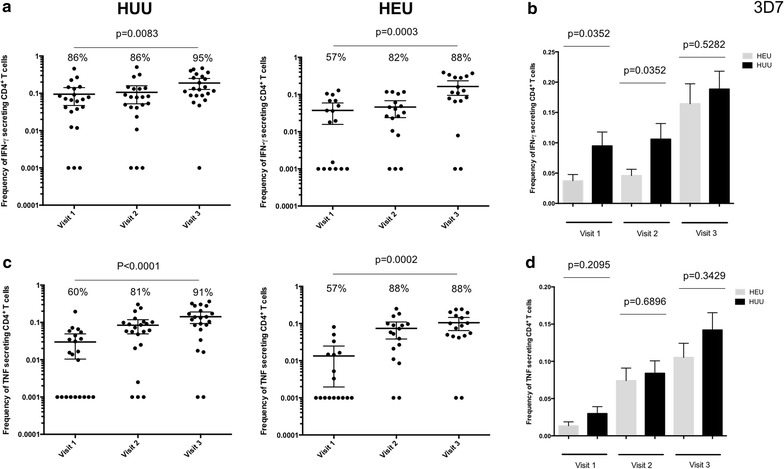
Frequency of PMA/Ionomycin stimulated CD4^+^ T cells in HEU and HUU children at different ages. **a** Total percentage of all IFN-γ cytokine producing CD4^+^ T cells was determined in HEU children and compared to HUU children at 6, 12 and 18 months of age. **b** Histogram comparing total percentage of all IFN-γ cytokine producing CD4^+^ T cells between HEU and HUU children overtime. **c** Total percentage of all TNF cytokine producing CD4^+^ T cells was determined in HEU children and compared to HUU children at 6, 12 and 18 months of age. **d** Histogram comparing total percentage of all TNF cytokine producing CD4^+^ T cells between HEU and HUU children overtime. X-axis represents time of visit. *Black horizontal bars* represent (**a, c**) mean with confidence intervals or (**b, d**) mean with SEM. Statistical significance was determined if *p* value was equal to or less than 0.05

To investigate whether there is delayed acquisition of *P. falciparum* antigen-specific CD4^+^T cell in HEU during CTX prophylaxis, the frequency IFN-γ- and TNF-producing CD4^+^T cells in whole blood following stimulation with antigens was determined (Fig. 2). It was found that in both HEU and HUU children there was acquisition of *P. falciparum* antigen-specific CD4^+^T cell with age (Fig. 3). Specifically, there was an increase in the proportion of responders with age, in both HEU and HUU children (Fig. 3a, c). There was also an increase in the frequency of *P. falciparum* antigen-specific CD4^+^T cells with age, in both HEU and HUU children (Fig. 3a, c). Interestingly, the frequency of *P. falciparum* antigen-specific CD4^+^T cells was lower in HEU children at 6 and 12 months in comparison to HUU children, which was the period the HEU children were on CTX prophylaxis (Fig. 3b, d). Upon CTX prophylaxis cessation, the frequency of *P. falciparum* antigen-specific CD4^+^T cells in HEU children normalized to levels seen in HUU age-matched controls (Fig. 3b, d).

**Fig. 4:**
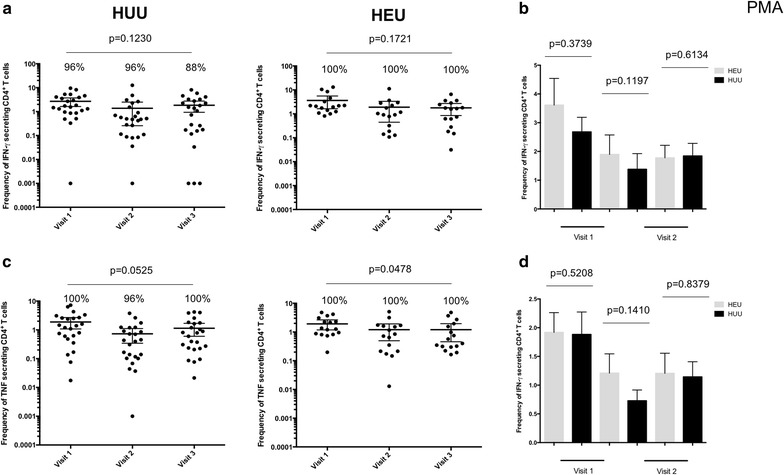
Frequency of *Plasmodium falciparum* antigen specific CD4^+^ T cells in HEU and HUU children at different ages. **a** Total percentage of all IFN-γ cytokine producing CD4^+^ T cells was determined in HEU children and compared to HUU children at 6, 12 and 18 months of age. **b** Histogram comparing total percentage of all IFN-γ cytokine producing CD4^+^ T cells between HEU and HUU children overtime. **c** Total percentage of all TNF cytokine producing CD4^+^ T cells was determined in HEU children and compared to HUU children at 6, 12 and 18 months of age. **d** Histogram comparing total percentage of all TNF cytokine producing CD4^+^ T cells between HEU and HUU children overtime. X-axis represents time of visit. *Black horizontal bars* represent (**a, c**) mean with confidence intervals or (**b, d**) mean with SEM. Statistical significance was determined if *p* value was equal to or less than 0.05

**Fig. 5:**
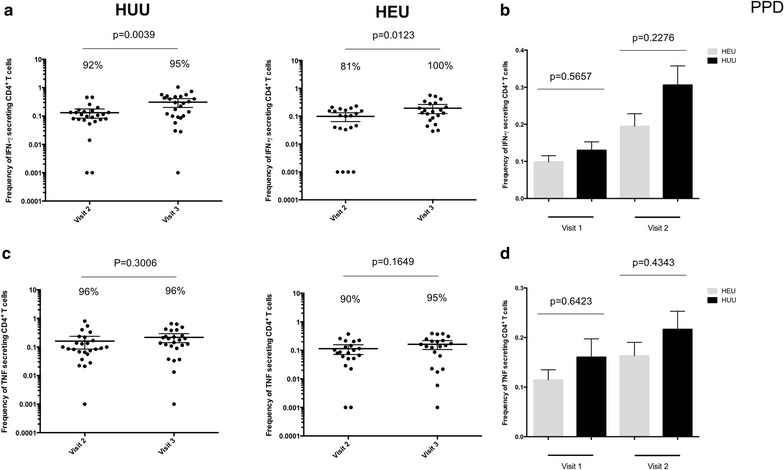
Frequency of mycobacterium antigen specific CD4^+^ T cells in HEU and HUU children at different ages. **a** Total percentage of all IFN-γ cytokine producing CD4^+^ T cells was determined in HEU children and compared to HUU children at 6, 12 and 18 months of age. **b** Histogram comparing total percentage of all IFN-γ cytokine producing CD4^+^ T cells between HEU and HUU children overtime. **c** Total percentage of all TNF cytokine producing CD4^+^ T cells was determined in HEU children and compared to HUU children at 6, 12 and 18 months of age. **d** Histogram comparing total percentage of all TNF cytokine producing CD4^+^ T cells between HEU and HUU children overtime. X-axis represents time of visit. *Black horizontal bars* represent (**a, c**) mean with confidence intervals or (**b, d**) mean with SEM. Statistical significance was determined if *p* value was equal to or less than 0.05

Next, it was hypothesized that if the lower frequency of *P. falciparum* antigen-specific CD4^+^T cells was due to generalized effects of CTX prophylaxis, then the frequency of mitogen-stimulated and antigen-specific CD4^+^ T cells in HEU children will also be lower than HUU age-matched controls. The results show that there was no difference in the frequency of mitogen-stimulated CD4^+^ T cells and the number of responders between HEU children and HUU age-matched controls at all ages (Fig. 4). It was also found that the frequency of mycobacterium-specific CD4^+^ T cells was not significantly different between HEU children and HUU age-matched controls at 12 and 18 months (Fig. 5). This suggests that the lower frequency of *P. falciparum* antigen-specific CD4^+^ T cells observed in HEU compared to HUU children was not generalized, but was specific to *P. falciparum*.

### Prevalence of asymptomatic and clinical malaria in HEU and HUU children

To determine whether the delayed acquisition of *P. falciparum* antigen-specific CD4^+^ T cells would result in higher prevalence of asymptomatic and clinical malaria in HEU children. The number of children with asymptomatic malaria at each scheduled visit was similar in both groups; 6 months (HEU 3 vs. HUU 6.5 %), 12 months (HEU 6.5 vs. HUU 0 %) and 18 months (HEU 6.7 vs. HUU 7.1 %). The number of HEU and HUU children with at least one episode of clinical malaria at any time of the follow up period was similar between HEU and HUU children (9.1 vs. 12.9 %). There was no evidence of increased prevalence of asymptomatic and clinical malaria in HEU children on CTX prophylaxis compared to HUU age-matched controls, but the study was underpowered to conclusively determine this clinical endpoint.

## Discussion

This study investigated the effect of CTX prophylaxis on the acquisition of *P. falciparum* antigen specific CD4^+^ T cells in HEU children. The study findings demonstrate that in the context of unaltered proportions of naïve and memory CD4^+^ T cell subsets and unimpaired CD4^+^ T cell IFN-γ and TNF producing function, the frequency of *P. falciparum* antigen-specific IFN-γ-producing CD4^+^ T cells was lower in HEU children on CTX prophylaxis compared to HUU age-matched controls. However, this normalized to levels seen in HUU children, 6 months upon CTX prophylaxis cessation.

Phenotypic alterations in the proportions of naive and memory T cell subsets have been observed in HEU children during early life [27, 28]. Consequently, alterations in the numbers or proportions of CD4^+^ T cell subsets may affect the capacity of antigen specific CD4^+^ T cell response to infections [29], leading to an increase in risk of infections in HEU children [30–32]. In this study, there was no difference in proportion of CD4^+^ T cell subsets in between HEU and HUU children from 6  to 18 months of age indicating that HEU children have normal CD4^+^ T cell phenotypes, contrary to earlier reports which were done mostly during the pre-ART era [28, 33].

The frequencies of *P. falciparum* antigen-specific IFN-γ-producing CD4^+^ T cells in HEU children during the period on CTX prophylaxis was lower in comparison to HUU age-matched controls. However, there was no differences in the mitogen-stimulation or Mycobacterium-specific CD4^+^ T cells. IFN-γ plays an important role in regulating the protective immune responses against blood stage malaria infection even in the absence of antibody response [34] and has been shown to be a potential marker of *P. falciparum* exposure [35]. Similar lower frequencies of *P. falciparum* specific CD4^+^ T cells were observed in Ugandan under-five children with lower prior malaria incidence compared to children with higher prior incidence[12]. This observation supports the idea that CTX prophylaxis reduces natural exposure to *P. falciparum*, resulting in a delayed acquisition of cell-mediated immunity against malaria, which ultimately may result in impaired development of *P. falciparum* antigen-specific antibody-mediated immunity [21].

In contrast to previous results in the same population [21], where antibody responses in HEU children remained low post-CTX prophylaxis reflecting delayed acquisition of antibody responses following CTX prophylaxis, the frequency of *P. falciparum* antigen-specific IFN-γ-producing CD4^+^ T cells normalized following cessation of prophylaxis and were comparable to HUU children by 18 months of age. Studies from experimental malaria challenge showed that even a single exposure to malaria induced robust CD4^+^ T cell responses producing IFN-y that could be detected 14 months post infection in the absence of further exposure[6]. These results might therefore explain the current observation that showed a significant increase in the frequency of IFN-γ producing CD4^+^ T cells in response to *P. falciparum* in HEU children after CTX prophylaxis suggesting different patterns in acquisition of humoral and T cell mediated immunity to malaria following CTX prophylaxis.

Due to the importance of the IFN-γ in immunity against malaria, it would be anticipated that delayed acquisition of *P. falciparum* antigen-specific IFN-γ-producing CD4^+^ T cells would lead to increase risk to asymptomatic and clinical malaria. Unfortunately, this current study could not gather enough clinical endpoints, as it was observed that the prevalence of parasitaemia and clinical malaria in both HEU and HUU children in the study was much lower than the estimated 27 %. This underpowered the study to conclusively show any clinical endpoint differences attributed to the CTX induced perturbation in *P. falciparum*-specific immunity between the two study groups. Nevertheless, the study reports an important observation showing delayed acquisition of *P. falciparum*-specific immunity in HEU children during CTX prophylaxis, which requires further investigation.

The choice of immune responses investigated in the study is not exhaustive, and the authors acknowledge that there are other mechanisms that are equally as important. CD4^+^ T cells producing other cytokines such IL-2, IL-10 and IL-4 alone or in combination with the studied cytokines have been shown to be associated with protection from malaria [6, 8, 11, 12]. However, IFN-γ and TNF are among the major cytokines shown to be associated with immunity against malaria, hence the study provides valid insight on the effect of CTX prophylaxis on antigen-specific CD4^+^ T cells immune responses. Secondly, although CD4^+^ T cells are an important producer of IFN-g, they are not the only cells that produce this important cytokine since macrophages, NK and B cells [36, 37] are also known to be a good source of this cytokine. Furthermore, it would have been ideal to have HEU children not on CTX prophylaxis or to have a subgroup of HEU children on CTX prophylaxis beyond 12 months, but this was deemed unethical and not justifiable within the study setting, hence the study had to be done in line with the national policy for Malawi that requires daily CTX prophylaxis to be given to all HEU children from 6 weeks to 12 months of age.

In conclusion, the study has shown that CTX prophylaxis is associated with delayed acquisition of *P. falciparum* antigen-specific CD4^+^ T cells in HEU children. This has potential to increase the risk of malaria infection following cessation of CTX prophylaxis, but this study was not able to conclusively address this hypothesis due to limitations in sample size and the duration of the follow-up period. Future studies should be based on a larger sample size and longer follow up durations to ascertain definitively the impact of CTX prophylaxis mediated delay in anti-malarial immunity on risk to clinical malaria. This is a public health dilemma that needs to be tackled.
